# Effect of a 10-Methacryloyloxydecyl Dihydrogen Phosphate-treated Bioceramic Sealer on the Bond Strength of an Endodontic Fiber Post: Multilayer Composite Disk Models and Ultra-highspeed Imaging Analysis

**DOI:** 10.3290/j.jad.b4997329

**Published:** 2024-02-21

**Authors:** Chi-Hung Chen, Kuan-Han Lee, Ching-Chih Wei, Po-Yen Lin, Wan-Chuen Liao, Chih-Wen Chi, Alex S.L. Fok, Yu-Chih Chiang

**Affiliations:** a Doctoral Student, School of Dentistry and Graduate Institute of Clinical Dentistry, National Taiwan University, Taipei City, Taiwan. Data acquisition and interpretation, drafted the manuscript, gave final approval and agreed to be accountable for all aspects of the work.; b Dentist, School of Dentistry and Graduate Institute of Clinical Dentistry, National Taiwan University, Taipei City, Taiwan; Department of Dentistry, Shin-Kong Wu Ho-Su Memorial Hospital, Taipei, Taiwan. Data interpretation and manuscript revision, gave final approval and agreed to be accountable for all aspects of the work.; c Engineer, Department of Mechanical Engineering, National Taiwan University of Science and Technology, Taipei City, Taiwan. Data acquisition and interpretation, drafted the manuscript, gave final approval and agreed to be accountable for all aspects of the work.; d Adjunct Assistant Professor, Department of Dentistry, School of Dentistry, National Yang Ming Chiao Tung University, Taipei, Taiwan. Data analysis and interpretation, gave final approval and agreed to be accountable for all aspects of the work.; e Doctoral Student, School of Dentistry and Graduate Institute of Clinical Dentistry, National Taiwan University, Taipei City, Taiwan. Drafted the manuscript, gave final approval and agreed to be accountable for all aspects of the work.; f Dentist, Department of Dentistry, National Taiwan University Hospital Hsin-Chu Branch, Hsin-Chu, Taiwan. data interpretation and critically revised the manuscript, gave final approval and agreed to be accountable for all aspects of the work.; g Professor, Minnesota Dental Research Center for Biomaterials and Biomechanics (MDRCBB), School of Dentistry, University of Minnesota, Minneapolis, MN, USA. data interpretation and critically revised the manuscript, gave final approval and agreed to be accountable for all aspects of the work.; h Professor, School of Dentistry and Graduate Institute of Clinical Dentistry, National Taiwan University, Taipei City, Taiwan; Division of Restorative and Esthetic Dentistry, Dental Department, National Taiwan University Hospital, Taipei City, Taiwan; School of Dentistry, Kaohsiung Medical University, Kaohsiung, Taiwan. Conception and design of the study, data acquisition, analysis, and interpretation and drafted and critically revised the manuscript, gave final approval and agreed to be accountable for all aspects of the work.; † Kuan-Han Lee and Chi-Hung Chen contributed equally to the work.

**Keywords:** bioceramic-based sealer, bond strength testing, fiber post, finite element analysis, multilayer composite-disk model, ultra-highspeed imaging

## Abstract

**Purpose:**

Multiple materials are found in the root canal after fiber-post cementation. The layer of a bioceramic-based (BC) sealer may affect the bond strength (σ_BS_) of the fiber post in the root canal. The purpose of this study was to employ multilayer composite-disk models in diametral compression to investigate whether the bond strength between a fiber post and root dentin can be increased by the application of a primer on the BC sealer.

**Materials and Methods:**

The multilayers of materials in the root canal required 3D finite-element (FE) stress analyses (FEA) to provide precise σ_BS_ values. First, BC sealer was characterized using x-ray powder diffraction (XRD) to determine when the sealer completely set and the types of crystals formed to select which primer to apply to the sealer. We selected a 10-methacryloyloxydecyl dihydrogen phosphate (10-MDP)-based primer to treat the BC sealer before post cementation. Ultra-highspeed (UHS) imaging was utilized to analyze the crack initiation interface. The obtained failure force was used in FE analysis to calculate σ_BS_.

**Results:**

UHS imaging validated the fracture interface at the post-dentin junction as FEA simulations predicted. σ_BS_ values of the fiber posts placed with various material combinations in the root canal were 21.1 ± 3.4 (only cement/ post), 22.2 ± 3.4 (BC sealer/cement/post) and 28.6 ± 4.3 MPa (10-MDP primer treated BC sealer/cement/post). The 10-MDP-treated BC sealer exhibited the highest σ_BS_ (p < 0.05).

**Conclusion:**

The multilayer composite disk model proved reliable with diametral compression testing. The presence of BC sealer in the root canal does not reduce σ_BS_ of the fiber post. Conditioning the BC sealer layer with 10-MDP primer before fiber-post cementation increases σ_BS_.

An intraradicular fiber post retains the restoration of an endodontically treated tooth due to the comparable elastic modulus to root dentin and the ability to bond to the root dentin via composite cement.^16^ However, the placement of a fiber post may not enhance fracture resistance of structurally compromised roots. Root canal sealers may also interfere with the bonding between the composite cement and the root dentin or fiber post.^21,29,32^ Clinically, root canal sealers can be classified according to their chemical content, i.e., zinc oxide eugenol-based, calcium hydroxide-based, glass-ionomer-based, resin-based, or bioceramic-based (BC) sealers. Non-resin-based sealers may not bond well with composite cement, particularly those containing eugenol, which interferes with the curing of composite cement.^25,33^ If the root-canal sealer interferes with polymerization or fails to achieve satisfactory bonding with the composite cement, the fiber post may loosen or dislodge, thus causing prosthesis failure, placing the tooth at increased risk of reinfection of the root canal system.^2,30,34^

BC sealers consist of calcium silicates, calcium phosphate monobasic, calcium hydroxide, and zirconium oxide.^28^ BC sealers easily adapt to the noncircular root-canal wall and fill the root canal space through the single-cone filling technique.^19^ Due to the bioactive and biocompatible properties of BC sealers, they have become more popular as a root canal sealer. Özcan et al^22^ reported that a calcium silicate-based sealer did not adversely affect the bond strength of the fiber posts cemented with self-adhesive composite cement. However, some studies reported that calcium silicate-based BC sealers negatively interfere with the bond strength of the fiber-post/resin-cement junction to root dentin.^21,32^ They investigated the bond strength of the fiber post to root dentin using the push-out test. However, differences in root canal geometries, fiber-post tapers, and indenter diameters may generate different test results.

Huang et al^12^ proposed a diametral compression test with composite disks to determine the interfacial bond strength between the intracanal post and dentin. Using the digital image-correlation technique, they validated that debonding initiated at and propagated along the post-dentin interface. Additionally, by using a finite element analysis (FEA), they further calculated the bond strength from the load at failure. The FEA model contained three major components: the outermost resin-composite layer to form a circular disk specimen, root dentin in the middle, and the fiber post at the center; however, the root canal sealer was not considered. Therefore, new models would be needed if a sealer layer exists at the root canal wall. Moreover, as mentioned above, there are both positive and negative arguments about the impact of BC sealer on the adhesion of composite cement.

The primary objective of this study was to develop multilayer composite disk models specifically designed for diametral compression. The rationale behind this endeavor was to assess the bond strength (σ_BS_) between the fiber post and root dentin in the presence of a BC sealer, with a secondary aim of investigating the potential influcence of applying a primer to the BC sealer on enhancing σ_BS_. To achieve these goals, we characterized the elastic modulus and Poisson’s ratio of the BC sealer to construct FEA models. These three-dimensional (3D) FEA models of the multilayer composite disks in diametral compression allowed analysis of the stress distribution, enabling the calculation of σ_BS_ based on the obtained failure load. An ultra-highspeed (UHS) camera was used to document the fracture process and validate the debonding initiation during compression. The null hypothesis posited that applying a primer to the BC sealer surface before fiber-post cementation would not significantly affect σ_BS_ when compared to cases in which no primer was applied.

## Materials and Methods

### Root Canal Preparation and Fiber-Post Cementation for Multilayer Composite Disks

In total, 24 single-root intact human mandibular premolars and maxillary central incisors were collected after obtaining informed consent from the patients (Institutional Review Board approval no. 201802078RINC). The teeth were all decoronated at the cementoenamel junction with a low-speed diamond saw (Isomet 1000, Buehler: Lake Bluff, IL, USA). An endodontic specialist prepared the root canal based on the Guide to Clinical Endodontics 6th edition of the American Association of Endodontists. A ProTaper System (Dentsply Maillefer; Ballaigues, Switzerland) was utilized to achieve a master apical file size up to F5 with 5% sodium hypochlorite irrigation. Post lengths measuring 10 mm were prepared with a RelyX Fiber Post and Drill kit (3M Oral Care; St Paul, MN, USA). Each endodontically prepared root portion was embedded in light-curing composite resin (Z250, 3M-Oral Care) in a custom-made 10-mm-diameter cylinder mold.

**Fig 1:**
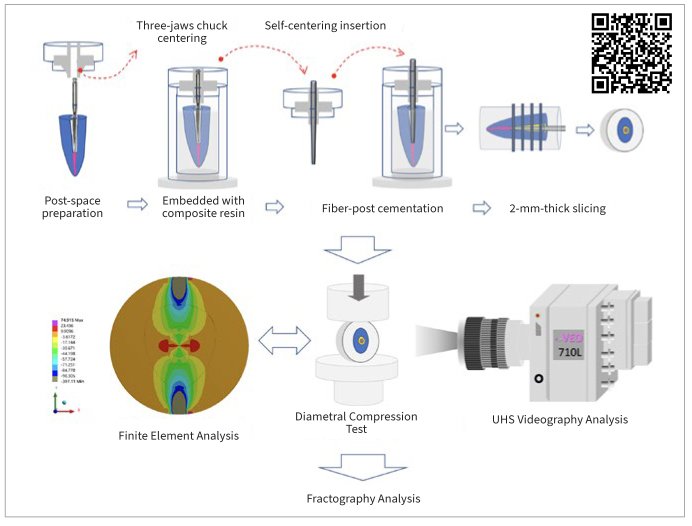
Workflow for positioning the drill and fiber post in the center of the prepared root canal space and setup for the diametral compression test. Bond strength (σ_BS_) was calculated via finite element analysis. Ultra-highspeed (UHS) videography analysis was simultaneously performed during the diametral compression test to record the fracture process and validate crack initiation.

The 24 teeth were divided into 3 groups for fiber-post cementation. To standardize the cementation procedure, the root canal was not filled with gutta-percha. For the “Sealer-Cement” group, after the post space was prepared with post drill #3, the BC sealer was placed in the post space, followed by seating with a Teflon-coated fiber post (RelyX Fiber Posts #2, 3M Oral Care). After 7 days, we removed the Teflon-coated fiber post and cemented the fiber post with U200 (RelyX U200 self-adhesive composite cement, 3M Oral Care). The space of the Teflon-coated layer on the fiber post was readied for the U200 (3M Oral Care) cement. A three-jaw chuck was used to accurately center the drill and the direction of the fiber-post insertion into the root canal (Fig 1). Two slices from the coronal portion of each root were obtained for the diametral compression test of multilayer composite disks. A similar procedure was applied for the “Sealer-Primer-Cement” group: a 10-MDP-containing primer (Clearfil Ceramic Primer Plus, Kuraray Noritake; Tokyo, Japan) was applied on the surface of the set BC sealer and then the fiber post was cemented. For the “Cement-Only” group (which served as the control), we prepared the post space with a post drill (3#) and then directly cemented the fiber post (#2) with U200 composite cement (without using BC sealer beforehand).

### Preparation of Multilayer Composite Disks

Each endodontically prepared root was embedded in a light-curing resin composite (Z250, 3M Oral Care) in a custom-made 10-mm-diameter cylindrical mold. The samples were randomly divided into 3 groups (N=8) for the 3 different cementation protocols. Each embedded root was sectioned into 2-mm-thick slices at the coronal root portion with a low-speed diamond saw (Isomet, Buehler; Lake Bluff, IL, USA) to obtain 2 multilayer composite disks (n=16 for each group), which were then stored at 100% humidity and 37°C for 24 h before testing. The sample size was calculated using G*Power 3.1.9.7 for Windows: effect size f = 0.47, α error probability = 0.050, power (1-β error probability) = 0.800. The compositions of the materials used in the study are listed in Table 1.

**Table 1:** Materials used in this study

Material	Manufacturer	Lot number	Composition (wt%)
Endosequence BC sealer	Brasseler; Lemgo, Germany	16004SP	Zirconium oxide (35.0-45.0%), tricalcium silicate (20.0-35.0%), dicalcium silicate (7.0–15.0%), calcium hydroxide (1.0–4.0%) Others: calcium phosphate monobasic, fillers and thickening agents
Panavia V5 Clearfil Ceramic Primer Plus	Kuraray Noritake; Tokyo, Japan	7T0037	3-methacryloxypropyl trimethoxy silane <5%, 10-methacryloyloxydecyl dihydrogen phosphate (10-MDP), ethanol (>80%)
RelyX U200 composite cement (Automix system)	3M Oral Care; St Paul, MN, USA	654051	Base: methacrylate monomers containing phosphoric acid groups, methacrylate monomers, initiators, stabilizers, rheological additives Catalyst: methacrylate monomers, alkaline fillers, silanated fillers, initiator components, stabilizers, pigments, rheological additives, zirconia/silica fillers
RelyXM Fiber Post	3M Oral Care	313261605	Pretensioned, parallel glass-fiber embedded in a composite resin matrix
Single Bond Universal	3M Oral Care	625715	10-MDP phosphate monomer, dimethacrylate resins, bis-GMA, HEMA, methacrylate-modified polyalkenoic acid copolymer, camphorquinone, filler, ethanol, water, initiators, silane
Filtek Z250 XT	3M Oral Care	N631653	Silane-treated ceramic, bis-GMA, bis-EMA, UDMA, TED-GMA, aluminum oxide

### Diametral Compression Test and Bond Strength (σ_BS_) Calculation and Ultra-highspeed Imaging Analysis

The diametral compression test was performed with a universal material testing machine (Instron 5566, Instron; Canton, MA, USA). The load cell’s capacity was 5 kN, and the crosshead speed was set to 0.5 mm/min. Meanwhile, a UHS camera (Phantom VEO-710L-Mono, Vision Research; Wayne, NJ, USA) was set up to record the diametral compression test. The frame rate was set at 200,000 fps. The serial UHS videographic images obtained during fracture were analyzed using ImageJ and Plot Profile software.^27^ The grayscale changes of UHS images were calculated before and after the fracture to analyze the detailed fracture process and identify where the crack was initiated. Then the stress distribution was determined within the multilayer composite disk under diametral compression using finite element (FE) analysis. The σ_BS_ between the fiber post and root dentin could then be calculated using the failure force.

### X-ray Powder Diffraction (XRD) Characterization of the Crystal Changes in the BC Sealer

**Fig 2:**
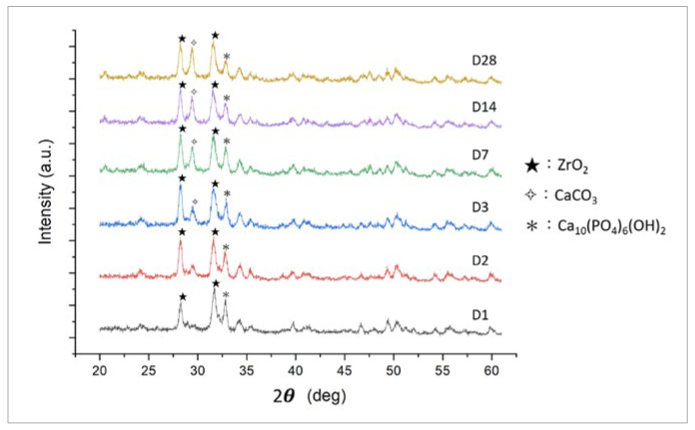
XRD analysis of the crystal layer on the BC sealer, which was obtained using PDF card no. 86-1450 for zirconium dioxide (ZrO_2_), no. 72-1243 for hydroxyapatite Ca_10_(PO_4_)_6_(OH)_2_ and no. 72-1937 for calcium carbonate (CaCO_3_). The most intense peaks for ZrO_2_ and hydroxyapatite were detected on the 1st day at 28.2 degrees and 32.9 degrees, respectively. Their intensities reached their maximum values and stabilized on the 3rd day. The intensity of the CaCO_3_ peak (29.4 degrees) increased over the first three days and reached the maximum value on the 7th day. After 7 days, all of the peaks displayed stable intensities.

For fiber-post cementation, the mature setting time of BC sealer on root dentin must be determined. To investigate the setting process, the crystal changes in the BC sealer on the sliced dentin disks were examined at various time points: after 1, 2, and 3 days, and 1, 2, and 4 weeks (Fig 2). The crystal phase of the BC sealer was identified via x-ray powder diffraction (XRD, X’Pert PRO, Philips; Eindhoven, the Netherlands), which was performed at 45 kV and 40 mA with a scanning rate of 2 degrees/min. The 2θ values ranged from 20 to 65 degrees.

### Finite Element Analysis (FEA)

**Fig 3:**
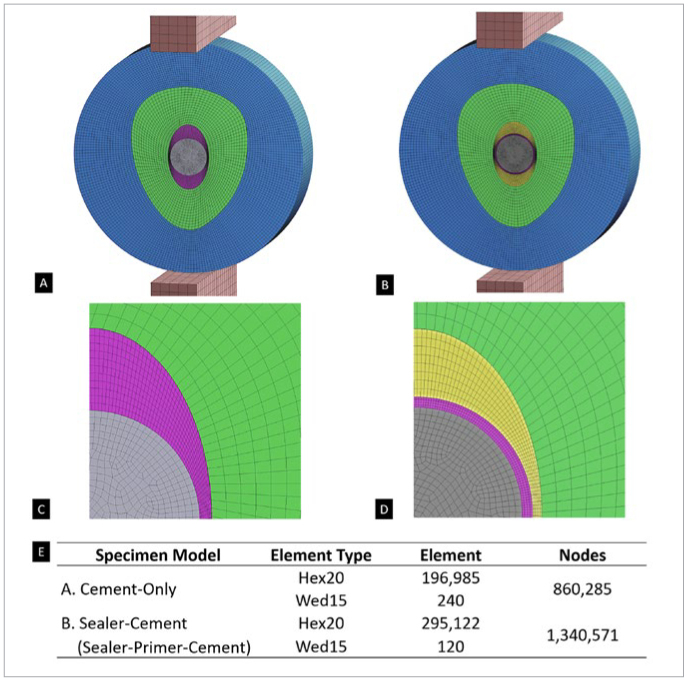
3D FEA models of (A) cement-only and (B) sealer-cement or sealer-primer-cement specimens. (C) and (D) High-magnification images of the central portions of the images in (A) and (B), respectively. The numbers of elements and nodes in each model are summarized in the table (E). Gray area: glass-fiber post. Pink area: composite cement (U200). Yellow area: BC sealer. Green area: root dentin. Blue area: resin composite. Hex20: hexahedral element with 20 nodes. Wed15: wedge element with 15 nodes.

Two 3D FEA models (Ansys 19.0, Ansys; Canonsburg, PA, USA) were constructed to simulate the different groups of multilayer composite disks (diameter: 10 mm, thickness: 2 mm) in diametral compression (Fig 3). The first model comprised a fiber post at the center surrounded by a layer of composite cement, followed by a layer of root dentin and then a layer of resin composite. This model served as the control, which was designated as the “Cement-Only” specimen (Figs 3A and 3C). The model for the “Sealer-Cement” specimen consisted of an additional layer of BC sealer applied on the root-canal dentin wall (Figs 3B and 3D). The samples that received an additional primer on the surface of the BC sealer, termed the “Sealer-Primer-Cement” specimen, were also represented by this second FEA model, as the very thin layer of primer was negligible.

Other than the fiber post, all materials were assumed to be homogeneous, isotropic, and linearly elastic. The properties are listed in Table 2. The fiber post was assumed to be transversely isotropic, with the longitudinal direction being the out-of-plane direction of the circular specimen. Since BC sealer is a relatively new material in dentistry, its mechanical properties have yet to be elucidated; first, therefore, its elastic modulus was measured as well as Poisson’s ratio for the FEA, as described in the following.

**Table 2:** Material properties/parameters required for FEA

Materials		Elastic modulus (GPa)	Poisson’s ratio	Ref.
Composite resin (Z250, 3M Oral Care)		14	0.31	1
Dentin		18.6	0.31	3
BC sealer		18.15	0.43	
Composite cement (U200, 3M Oral Care)		6.6	0.3	4
Fiber post	Longitudinal	37	0.34	2
	Transverse	9.5	0.27	

1. Chung SM, Yap AUJ, Koh WK, Tsai KT, Lim CT. Measurement of Poisson’s ratio of dental composite restorative materials. Biomaterials 2004;25:2455-2460. 2. Lanza A, Aversa R, Rengo S, Apicella D, Apicella A. 3D FEA of cemented steel, glass and carbon posts in a maxillary incisor. Dent Mater2005;21:709-715. 3. Peyton FA, Mahler DB, Hershenov B. Physical properties of dentin. J Dent Res 1952; 31:366-370. 4. RelyX™ U200 Technical Data Sheet, 3M Oral Care. https://multimedia.3m.com/mws/media/742338O/relyx-u200-technical-data-sheet-2-clicker-and-automix-syringe.pdf

The elastic modulus and Poisson’s ratio of the BC sealer were calculated by measuring its longitudinal and transverse ultrasonic wave velocities in addition to its density (Table 2). The BC sealer was placed into a plastic mold (inner diameter: 14.3 mm, height: 5.3 mm) and then stored at 37°C and 100% humidity for 7 days until setting (according to XRD identification, Fig 3). An ultrasonic transmitter (DPR300, JSR Ultrasonic; Pittsford, NY, USA) was used to measure the longitudinal and transverse ultrasonic wave velocities of the BC sealer, prior to which the density of the set BC sealer was calculated as 2.34 g/cm^3^. The formulae^23^ for calculating the elastic modulus and Poisson’s ratio of the BC sealer material are shown below.

The longitudinal ultrasonic wave velocity was calculated according to the formula:


$$C_{L}=\sqrt{\frac{E\;(1-\nu )}{\rho \;(1+\nu )\;(1-2\nu )}}$$

Eq. (1),

the transverse ultrasonic wave velocity was calculated using the formula:


$$C_{S}=\sqrt{\frac{E\;}{2\rho \;(1+\nu )\;}}=\sqrt{\frac{G}{\rho }}$$

Eq. (2),

where E is Young’s modulus (or the modulus of elasticity), ν is Poisson’s ratio, ρ is the density, and G is the shear modulus. Thus,


$$\nu =\frac{1-2{\left(\frac{C_{S}}{C_{L}}\right)}^{2}}{2-2{\left(\frac{C_{S}}{C_{L}}\right)}^{2}}$$

Eq. (3),

and


$$E=2\rho C_{S}^{2}\;(1+\nu )$$

Eq. (4).

The two steel compression platens used to apply the compression force were simulated. The bottom of the lower platen was fully fixed, and the compression force was introduced from the top of the upper platen. To ensure uniaxiality, the four vertical sides of the platens were constrained in their respective normal horizontal directions. The mesh was constructed with 20-node quadratic hexahedral elements and 15-node quadratic triangular prismatic elements, as shown in Fig 3E. The mesh was refined until changes in the stress results were less than 5%. To analyze the stress at the interfaces between the materials, a cylindrical coordinate system was used to allow the radial stress and hoop stress to be determined.

### Fractographic Analysis

The fracture surfaces and the elemental composition of the representative specimens were examined by using field-emission scanning electron microscopy and energy-dispersive x-ray spectroscopy (FE-SEM/EDS, Nova NanoSEM 230; Hillsboro, OR, USA). All specimens were sputter-coated with gold for 90 s at 20 mA after serial drying (Quorum Q150R S, Quorum Technologies; Nottingham, East Sussex, UK).

### Statistical Analysis

The obtained σ_BS_ data for the four groups were analyzed using one-way ANOVA and Scheffe’s post-hoc test (SPSS version 21.0 IBM; Armonk, NY, USA). The significance level was set at α = 5% for all statistical analyses.

## Results

### Stress Distribution Within the Multilayer Composite Disk under Diametral Compression

**Fig 4:**
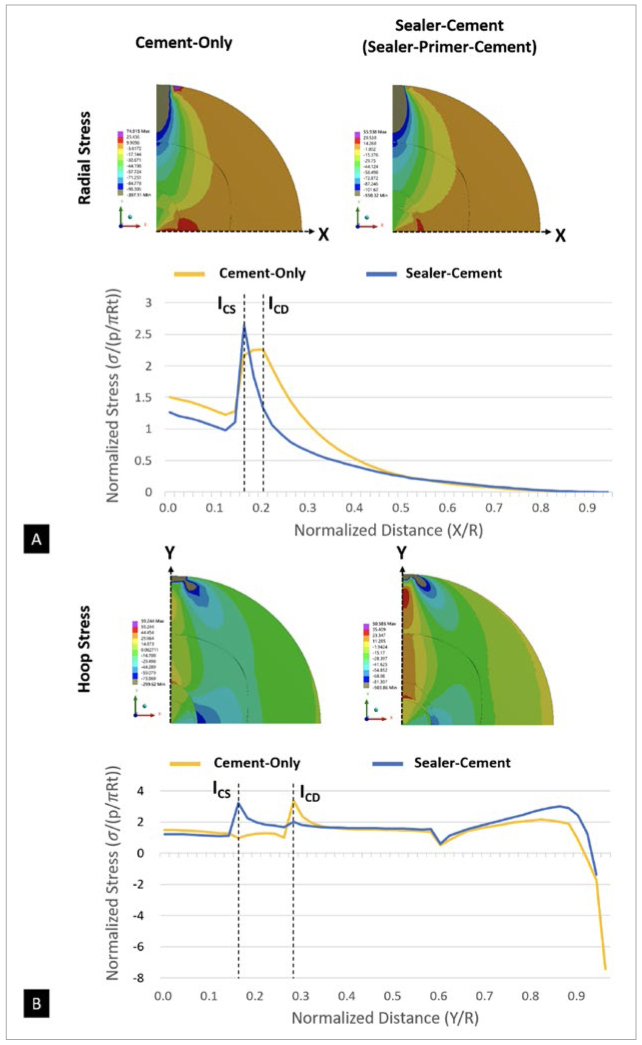
Stress distributions of composite disks under diametral compression. (A) Radial stress distribution and normalized radial stress along the x-axis of the two models. (B) Hoop stress distribution and normalized hoop stress along the y-axis of the two models. I_CS_ and I_CD_ indicate the cement-sealer and cement-dentin interfaces, respectively.

The FEA results for all specimens are shown in Fig 4. In the cement-only specimens, the highest tensile radial stress occurred at the cement-dentin interface (Fig 4A), and the highest tensile hoop stress (Fig 4B) was observed in the dentin region that was adjacent to the cement-dentin interface. In the sealer-cement and sealer-primer-cement specimens, the highest tensile radial stress occurred at the cement-sealer interface (Fig 4A), and the highest tensile hoop stress (Fig 4B) was observed in the cement that was adjacent to the cement-sealer interface. Due to the fact that the stresses were proportional to the load, it was possible to assess which component was more likely to fail first with increasing loads if the respective strength of the components was given. The diametral compression test demonstrated that: in the cement-only group, debonding would be initiated along the cement-dentin interface; in the sealer-cement and sealer-primer-cement groups, debonding would be initiated along the cement-sealer interface. The results for interfacial debonding were validated by performing UHS videography analysis.

### σ_BS_ and Ultra-highspeed (UHS) Videography

**Fig 5:**
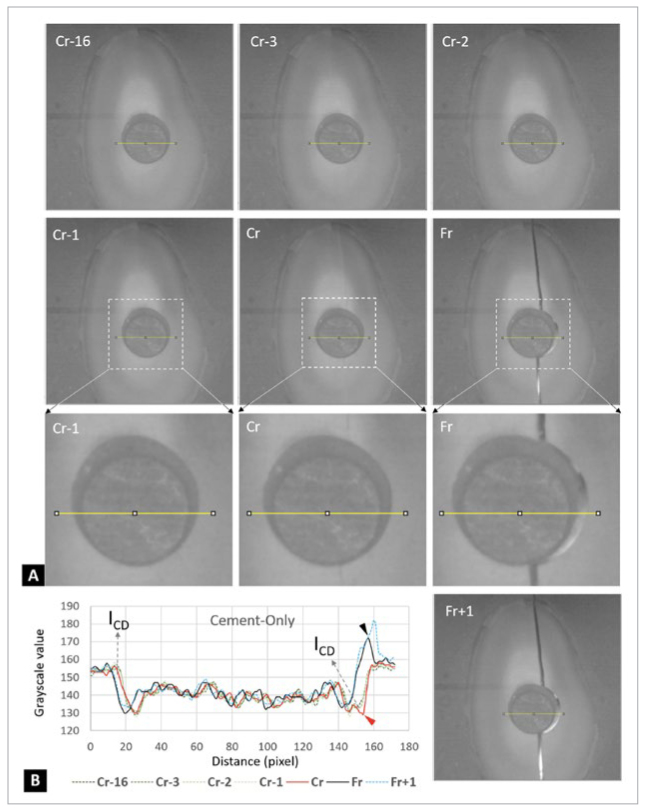
Serial UHS images of the diametral compression test of the cement-only specimen. (A) Cr-16 indicates the 16th frame before the crack was detected (Cr); Cr-3 indicates the 3rd frame before Cr. Cr-2 and Cr1 follow the same analogical pattern as described above. Fr indicates a complete fracture. Fr+1 indicates the frame after complete fracture. From Cr-16 to Fr+1, 19 frames were captured in 9.5 x 10^-5^ s, which shows the detailed fracture process during the diametral compression test. The yellow lines in each graph indicate where we analyzed the changes in grayscale values, which are summarized in the (B) Plot Profile. (B) The red arrowhead indicates where the crack started; this occurred at the interface of the composite cement and root dentin (I_CD_). The black arrowhead indicates that the specimen completely fractured and shifted to the sides. Pixel = 12 µm.

**Fig 6:**
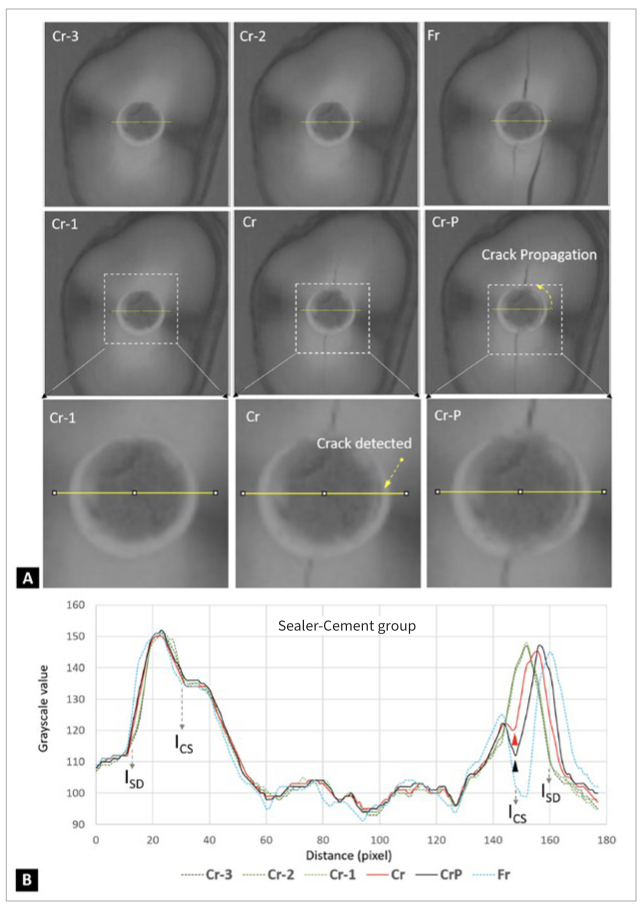
Serial UHS images of the diametral compression test for the sealer-cement specimen. (A) Cr-3 indicates the 3rd frame before the crack was detected (Cr). Cr-2 and Cr-1 follow the same analogical pattern as described above. Cr-P indicates crack propagation before a complete fracture (Fr). (B) Plot profile analysis of yellow line regions showed the changes in grayscale values of serial UHS images during the fracture process. The red arrowhead indicates that the crack was detected in the beginning, which occurred at the interface between cement and sealer (I_CS_). The black arrowhead indicates that the cracks in the specimen continued to propagate, and the specimen had not yet been completely fractured. I_SD_: interface between sealer and dentin. Pixel = 12 µm.

Figure 5A shows the cement-only specimen during the diametral compression test, as recorded by the UHS camera. The changes in grayscale values (Fig 5B) indicate that the crack was initiated at the interface of composite cement and root dentin (I_CD_), which was also predicted by FEA (Fig 4). For the sealer-cement group (Fig 6), serial UHS images of the diametral compression test revealed a crack along the central vertical diameter and around the interface between the BC sealer and the composite cement (Cr and Cr-P frames). The analysis of changes in grayscale values (Fig 6B) confirmed that the fracture was initiated at the stress concentration area, as predicted by FEA, i.e., the cement-sealer interface (I_CS_, Fig 4).

**Fig 7:**
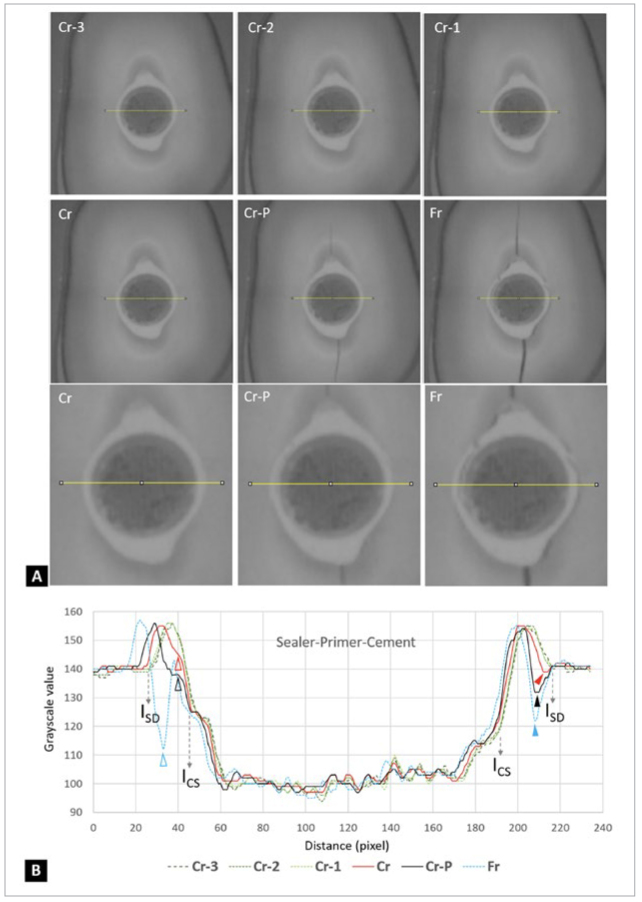
Serial UHS images of the diametral compression test for the sealer-primer-cement specimen. (A) Cr-4 indicates the 4th frame before the crack was detected (Cr). Cr-2 and Cr-1 follow the same analogical pattern as described above. Cr-P indicates crack propagation before a complete fracture (Fr). (B) Plot profile analysis showing the changes in grayscale values of serial UHS images during the fracture process. The red arrowhead indicates that the crack was detected in the very beginning of the process, which occurred at the interface between sealer and dentin (I_SD_). The black arrowhead indicates that the crack in the specimen continued to extend, while the specimen had not yet been completely fractured. The blue arrowhead indicates that the specimen was completely fractured and shifted to the left side by approximately 40 µm. I_CD_: Interface between cement and sealer. Pixel = 12 µm.

Figure 7A showed the serial UHS images of the diametral compression test for the sealer-primer-cement specimen. When combined with the grayscale value changes (Fig 7B), the serial images illustrated that the crack (solid red arrowhead) may have originated near one of the lateral sides of the sealer-dentin interface (I_SD_), after which the crack extended to both the top and bottom halves of the interface before rapid fractures occurred along the vertical diameter. Interfacial debonding may also have originated within the sealer region (hollow red arrowhead, Fig 7B) and deviated into the cement-sealer interface (I_CS_) prior to the final fracture. The debonding process prior to the catastrophic fracture spanned less than 10 frames, i.e., approximately 5 x 10^-5^ s.

Based on the above observations and the FEA results, the final failure loads were used to calculate the σ_BS_ values for the different groups, as shown in Table 3. The cement-only (21.1 ± 3.4 MPa) and sealer-cement (22.2 ± 3.4 MPa) groups had significantly lower σ_BS_ values than the sealer-primer-cement group (28.6 ± 4.3 MPa), with p < 0.05. The difference in bond strength between the cement-only and sealer-cement groups, however, was not statistically significant.

**Table 3:** Interfacial bond strengths for fiber posts under different conditions in the root canal

Test groups (N=16)	Mean ± SD (MPa)	Coefficient of variation (%)
Cement-Only	21.1 ± 3.4^a^	16.3
Sealer-Cement	22.2 ± 3.4^a^	15.1
Sealer-Primer-Cement	28.6 ± 4.3^b^	15.0

Means within a column followed by the same superscript letter are not significantly different at an alpha level of 5% via Scheffe’s test. The overall interfacial bond strength of the sealer-primer-cement group was significantly higher than that of the other groups (p< 0.05). All coefficients of variation were less than 20%.

### Fracture Surface Analysis

**Fig 8:**
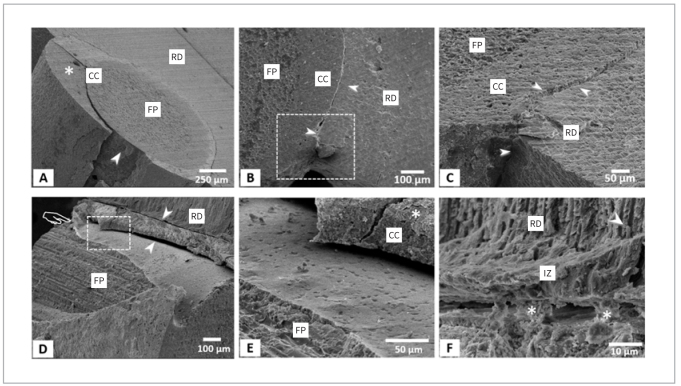
Fracture analyses by FE-SEM. (A) An example from the Sealer-Cement group. The arrowhead indicates the location of fracture initiation, which is the interface between the BC sealer (*) and composite cement. The fiber post (FP) and composite cement (CC) show an intact bond. (B) A representative specimen from the Cement-Only group. The arrowheads indicate the crack interface between the RC and root dentin (RD). (C) Higher magnification and tilted observation of the boxed area in (B). (D) A representative specimen from the Sealer-Primer-Cement group in which the CC achieved good adhesion with the BC sealer. The catastrophic fracture pulled out part of the coating of the fiber post (indicated by pointer) and exposed its fibers. Gaps existed between the RD and BC sealer and between the RC and FP (indicated by arrowheads). (E) Higher magnification of the boxed area in (D) shows good adhesion between the BC sealer (*) and CC. (F) Another specimen from the Sealer-Primer-Cement group in which the BC sealer and RD wall formed an integrated zone (IZ) and crystal tags (arrowhead), which may have helped to resist fracture. Fragments (asterisks) bridging the fracture gap between the BC sealer and IZ can be seen.

The FE-SEM images of the fracture surface are shown in Fig 8. The cement-post interface remained intact in the sealer-cement group (Fig 8A) and cement-only group (Figs 8B and 8C). For the sealer-primer-cement group, composite cement and the BC sealer can usually maintain a tight bond with the use of a 10-MDP primer (Fig 8E). In some specimens, the BC sealer can be found to form a tight junction with the root dentin to resist fractures (Fig 8F). In some specimens, we also observed that part of the composite cement was peeled off on the fiber post, and the fiber filaments were exposed (Fig 8D).

**Fig 9:**
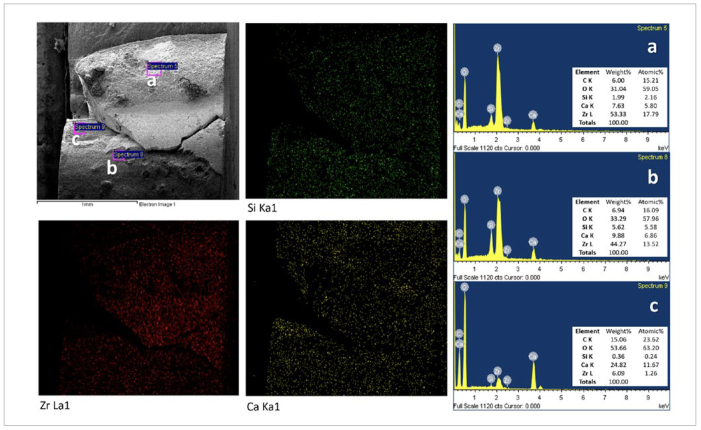
A representative Sealer-Primer-Cement specimen used for EDS mapping of the fracture surface. The red map shows the distribution of Zr and corresponding areas covered by the BC sealer. The BC sealer attached to the root dentin showed a high Zr intensity (region a). The BC sealer remained in most areas on the fiber post (region b), except for region c (less Zr signal).

EDS mapping of the fracture surface of a sealer-primer-cement specimen (Fig 9) confirmed that most BC sealers can be tightly bonded to the composite cement on the fiber post.

## Discussion

In this study, we employed a diametral compression test on multilayer composite disks to investigate the σ_BS_ between the endodontic fiber post and BC sealer based on the modified Brazilian disk test.^4,13^ According to the UHS images, the cracking started at the cement-sealer interface at the intersection with the horizontal diameter, i.e., the point of maximum interfacial normal stress, and then deflected into the sealer-dentin interface before extending along the vertical diameter. These findings were also supported by the SEM fractography results. Based on the FEA-predicted maximum tensile stress at the interface where the fracture initiated, σ_BS_ was calculated by applying the failure load from the diametral compression test. Along with loading and UHS analyses, the results indicated that the entire specimen fractured almost immediately at the interface between fiber post and root dentin. Consequently, the null hypothesis was rejected, indicating that conditioning the BC sealer layer with a 10-MDP primer before fiber-post cementation would lead to an increase in σ_BS_ between the fiber post and root dentin.

In dentistry, numerous methods are available for testing the bond strength of root canal filling materials (or posts) to root dentin. These include the microtensile test, pull-out bond strength test, and push-out/punch shear bond strength test.^6,9,35^ However, these tests have shortcomings; for example, the microtensile bond strength test produces a high number of premature sample failures.^13,31^ The push-out bond-strength test was developed to provide a more uniform stress distribution across the post-dentin interface than the conventional shear bond strength test,^13,31^ but the bond strength measured is affected by the friction between the materials.^7,13,17^ When these test specimens are stored in water or subjected to aging, the frictional effect would be more pronounced because of hygroscopic expansion of the materials, especially when self-adhesive composite cements are used.^15,24^

In our study, the σ_BS_ obtained from the diametral compression test of multilayer composite disks revealed much lower coefficients of variation (average of 16.4% among all of the groups) than those obtained using the microtensile bond strengthtest (85%) or the micropush-out bond strength test (55%),^31,36^ consistent with the findings reported by Huang et al.^13^ Using a UHS camera, the fracture process at the interface between the fiber post and dentin was visualized in real time during the diametral compression test. To investigate the crack initiation and propagation processes, we calculated and tracked the grayscale values of the post-cement-sealer-dentin interface at the intersection of the horizontal diameters in the series of images obtained by the UHS camera under the diametral compression test. The recorded images could be repeatedly examined to identify the location of the crack initiation. However, it was not always possible to observe crack changes in UHS images with the naked eye alone. For example, in Fig 7A, it is difficult to distinguish the interface changes between Cr and Cr-1 UHS frames only through digital magnification and the naked eye. Thus, it is advantageous to examine the interface changes by analyzing the changes of the grayscale values (Fig 7B). This information helps us to more clearly examine the fracture process and mechanisms of the multilayer composite disks under the diametral compression test, as well as to verify the results of the finite element analysis. Moreover, SEM can provide extremely detailed images to evaluate the fracture surface; however, real-time imaging is not possible, and is destructive and time-consuming.^11^

One of the main functions of root canal sealers is to form a bond between the core of the filling material and the root canal wall.^1^ In this study, the BC sealer was found to form an integrated seal between the post and the root dentin wall. Even after the fracture, a thin layer of the resulting crystallized material remained attached to the root dentin wall (Fig 8). The tag-like structures formed in dentinal tubules has also been suggested to contribute to the bond strength to root dentin.^10^ In addition to bioinert ZrO_2_, this BC sealer also contained bioactive ceramics such as tricalcium silicate (20.0–35.0%) and dicalcium silicate (7.0–15.0%) (Table 1).^7^ This calcium-silicate-based material has been shown to produce mineralization and precipitate directly onto the surrounding hard tissue (i.e., the root dentin and dentinal tubules, see Fig 8F). This precipitate, which has a crystalline structure with a chemical composition similar to that of tooth apatite, resulted in chemical bonding to root dentin.^5^ Together, this combination may form a hydroxyl-carbonate apatite that maintains the quality and stability of the bond.^14^ Consequently, an integrated zone (IZ) formed between the BC sealer and root dentin, in which tags penetrated into the dentinal tubules to achieve micromechanical interlocking. Some of the fragments found at the fractured interface still maintained contact with each side, indicating that the integrated zone may have helped resist fracture through crack bridging. Most of the specimens in the sealer-cement group fractured at the sealer-cement interface (Fig 6). However, this does not mean that the bond between the BC sealer and composite cement was weaker than that between the BC sealer and root dentin. The interfacial tensile stress was concentrated at the sealer-cement interface based on FEA. The same can be said about the cement-only group, in which the fracture mostly occurred at the interface between dentin and composite cement (Fig 5). However, in this case, the interfacial tensile stress at the cement-dentin interface was similar to that at the post-cement interface. Thus, the composite cement adheres more strongly to the fiber post than to dentin. This is unsurprising given that the fiber post comprised fibers coated with resin and thus must have bonded well with the resin luting cement.

In the sealer-primer-cement group, in which a primer was applied to the sealer before post cementation, the fractures mostly occurred at the interface between sealer and root dentin or near the sealer-cement interface (Fig 6D). Due to the fact that the maximum interfacial tensile stress still occurred at the sealer-cement interface (Fig 4), the primer must have made the cement-sealer interface much stronger than the sealer-dentin interface. Clearfil Ceramic Primer Plus (Kuraray Noritake) contains a phosphate-based functional monomer, which is known as 10-MDP. This molecule forms chemical bonds with the calcium in hydroxyapatite and CaCO_3_^8^ and interacts with ZrO_2_ to form a stable hydrogen bond between the P=O and Zr-OH groups.^3, 20, 37^

The XRD results revealed that the set BC sealer was rich in ZrO_2_, hydroxyapatite, and CaCO_3_ crystals. Therefore, we postulate that the 10-MDP primer helps the composite cement to bond well with the BC sealer due to the resulting formation of monomer-Zr and monomer-Ca salts.

Many factors influence the prognosis of restorations in endodontically treated teeth; for instance, loose posts and unrestorable root fractures are the most common causes of failure. Loosening of the interface between the fiber post and the tooth can significantly increase the stress in the tooth, which was confirmed by finite element analysis, thereby increasing the risk of root fracture.^26^ After loosening, the post-core-crown-tooth complex will no longer be able to sustain occlusal loads as a single unit. Adequate bond strength between fiber post and root dentin with a composite crown may increase the fracture resistance and reduce the risk of unrestorable fracture.^18^ Therefore, ensuring adequate bond strength between the fiber post and root dentin is critical to the success of restorations of endodontically treated teeth.

The present study conducted only debonding tests with freshly prepared samples under static loading, whereas most clinical failures are caused by cyclic fatigue and aging. The effects of shrinkage and hygroscopic expansion in the adhesive cement have also not been fully considered. Despite these limitations, the present study establishes the feasibility of using the multilayer composite disk in diametral compression to determine the bond strength between intraradicular fiber posts and root dentin. Further research will be conducted to determine the effect of cyclic loading and aging on the interfacial bond strength.

## Conclusion

The multilayer composite disk model with diametral compression testing can be applied to measure the interfacial bond strength between a fiber post and root dentin in the presence of a bioceramic sealer. An ultra-highspeed camera validated the simulation result of the finite element analysis by providing more detailed and dynamic information about the fracture initiation and propagation. The presence of BC sealer in the root canal did not impair the bond strength of the fiber-post cementation. In clinical practice, surface treatment of BC sealer with 10-MDP primer before fiber-post cementation is to increase the interfacial bond strength.

## Clinical relevance:

Surface treatment of BC sealer with 10-MDP primer before fiber-post placement can increase the bond strength between a fiber post and root dentin.
